# A Rare Case of Mandibular Mucormycosis in a Post-COVID-19 Patient

**DOI:** 10.7759/cureus.28216

**Published:** 2022-08-20

**Authors:** Naqoosh Haidry, Kranti Bhavana, Peeyush Shivhare, Vivek Kumar, Aiswarya Vaidyanathan

**Affiliations:** 1 Department of Dentistry, All India Institute of Medical Sciences, Patna, IND; 2 Department of Otolaryngology, All India Institute of Medical Sciences, Patna, IND

**Keywords:** amphotericin b, rhino-orbito-cerebral-maxillary, covid, mandible, mucormycosis

## Abstract

Mucormycosis or black fungus is one of the most lethal, progressing, and rapid form of deep fungal infections in humans which is caused by group of molds called mucormycetes. It is called black fungus infection due to black nasal discharges and black patches around nose found in the initial stage. The most common variety is rhino-orbito-cerebral-maxillary, although there are also pulmonary, gastrointestinal, cutaneous, and disseminated variations. In recent years, mucormycosis has become increasingly prevalent in immunocompromised individuals, with coronavirus disease 2019 (COVID-19) infection and associated consequences as the primary source of the cause. Rhino-orbito-cerebral-maxillary variety usually involves the nose, paranasal sinuses, brain, and maxilla but the involvement of mandibular bone is exceedingly rare. In this case report, we present a case of a 59-year-old male affected with mandibular mucormycosis in post-COVID scenario which is quite rare situation.

## Introduction

Mucormycosis is a deep fungal infection caused by a saprophytic fungus that is a member of the Phycomycetes phylum and is characterized by deep, fulminant, and acute fungal infection. Although Baker first used the name in 1957, Paltauf initially reported mucormycosis, also known as zygomycosis/Phycomycosis, in 1885 [1]. Immunosuppression related to cancers like leukemias and lymphomas, uncontrolled diabetes (particularly cases with diabetic ketoacidosis), renal failure, organ transplant, cirrhosis, burns, protein-energy malnutrition, long-term corticosteroid and immunosuppressive therapy, and AIDS are risk factors for mucormycosis. Mucormycosis can exist in different forms such as pulmonary, gastrointestinal, disseminated, and rhinocerebral. Rhinocerebral being the most prevalent form, involve paranasal sinus (mainly maxillary sinus), orbit, brain facial bones, and jaws [2]. This entity is very well-known for angioinvasion and tissue necrosis. Affected structures gradually experience tissue ischemia and necrosis as a result of the fungal hyphae invading the endothelium and causing thrombosis and infarctions [3].

During the second coronavirus disease 2019 (COVID-19) wave in India, this crippling illness received a lot of media attention. Numerous cases of post-COVID mucormycosis have been reported, primarily as a result of (a) uncontrolled diabetes with diabetic ketoacidosis; (b) using immunosuppressive medications such as tocilizumab, baricitinib, and tofacitinib; (c) cytokine storm; (d) increased free serum iron level (due to transferrin and ferritin glycosylation, serving as a substrate for the fungus); (e) increased host endothelium glucose regulator protein (GRP78) activity and its association with CotH3 (spore coat protein-ligand); and (f) endothelial damage and free radical injury lymphocytopenia [4]. Thus, the etiopathogenesis of mucormycosis linked with COVID-19 appears to be complex, with a number of variables interacting to cause and advance the disease.

The dramatic increase in mucormycosis cases in the Indian subcontinent following the second wave of SARS-CoV-2 infection has drawn attention to this potentially fatal condition. Due to the black color of diseased and necrotic tissue, the name "black fungus" is frequently used to characterize this illness. During the most recent wave of the pandemic, several cases of maxillary involvement in rhino-orbito-cerebral mucormycosis have been described, but relatively few cases have included the mandible. We now describe a mandibular instance of post-COVID mucormycosis. The case was first treated for mucormycosis of the maxillary sinus but after 2.5 to three months, the jaw became involved.

## Case presentation

**Figure 1:**
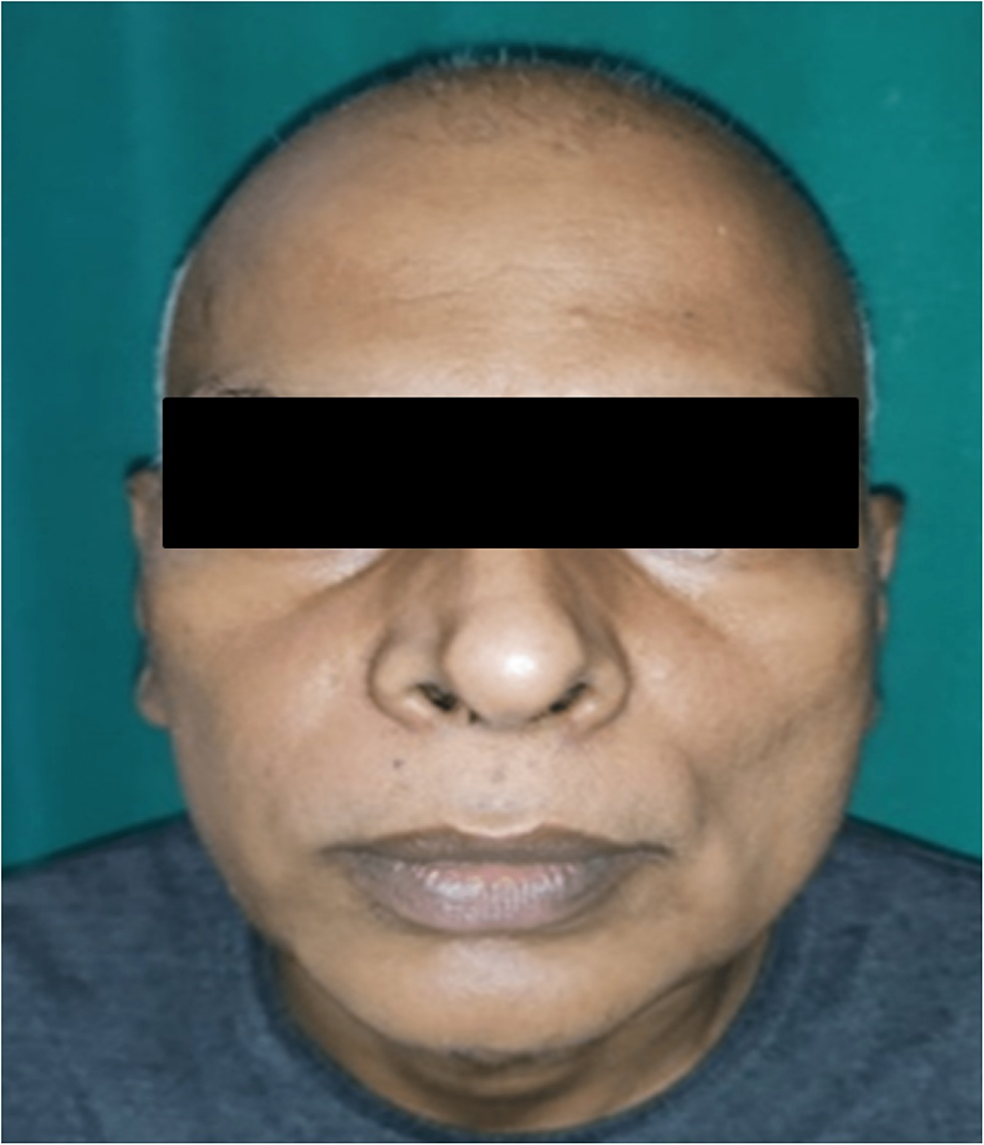
Extraoral view showing no significant pathology or obvious asymmetry.

**Figure 2:**
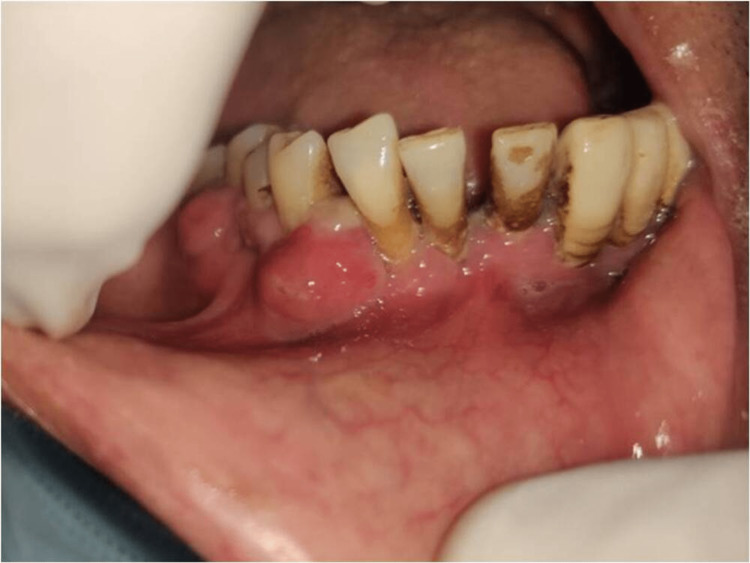
Intraoral view showing gingival swelling of right side.

We describe a 59-year-old man who visited the Department of Dentistry at All India Institute of Medical Sciences (AIIMS), Patna, with the primary complaint of gum swelling and teeth mobility on lower right side of mouth for 15-20 days. A COVID-19 infection had already occurred. A diagnosis of rhino-orbito-cerebral mucormycosis and the discovery of diabetes three months later led to endoscopic surgical debridement of devitalized tissue in the turbinates, maxillary sinus, and anterior and posterior ethmoid. The patient was asymptomatic and underwent routine follow-up at the Department of ENT, AIIMS, Patna, for the following 2.5 months. However, during that time, he experienced gingival swelling and tooth mobility on the lower right side of the oral cavity, and he was subsequently referred to the department of dentistry. Gingival swelling was discovered during a clinical examination in the 43-46 area (Figures 1, 2).

**Figure 3:**
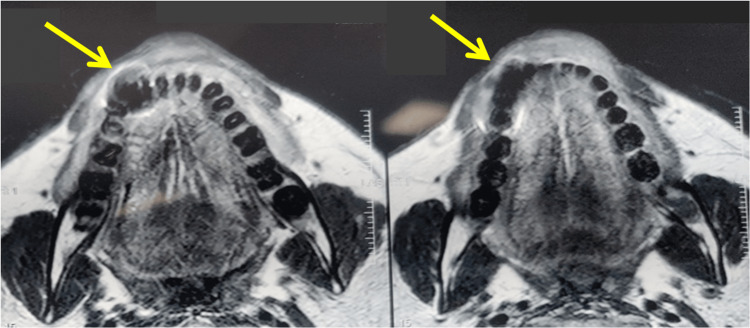
MRI showed a hypointense area of 42, 43, and 44 regions.

**Figure 4:**
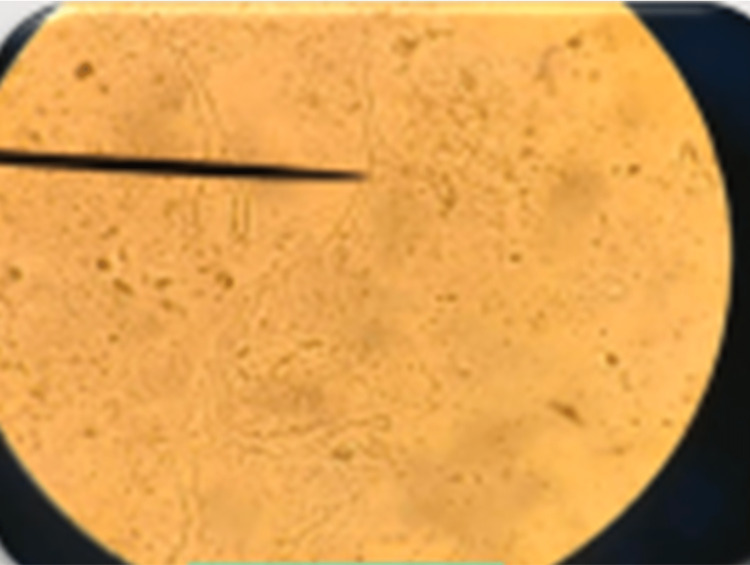
Mucor infection in the mandible verified by a KOH mount. KOH: potassium hydroxide

The impacted teeth had grade 2 mobility and the gingival surface was red, erythematous, and irritated. There was also pus discharge. The patient was informed that the afflicted area of the MRI showed a hypointense area of 42, 43, and 44 regions (Figure 3). The existence of a mucor infection in the mandible was verified by a KOH mount and biopsy of the gingival tissue and a portion of the alveolar bone performed under local anesthesia (Figure 4).

**Figure 5:**
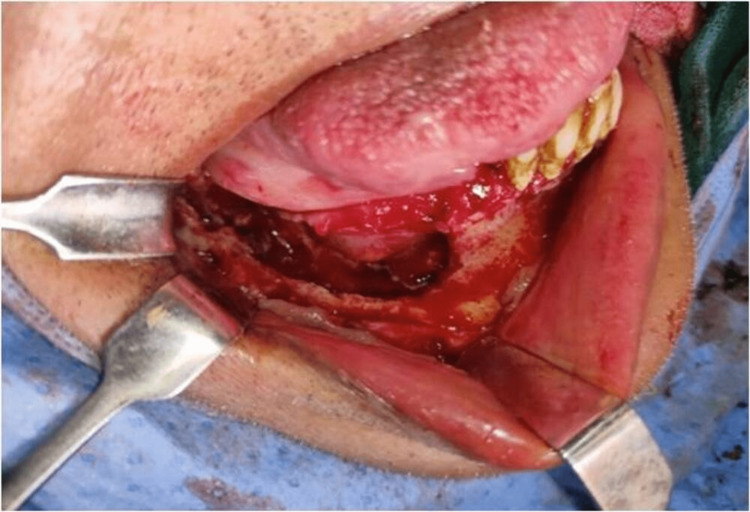
Intraoral operative procedure - surgical debridement of right mandible.

**Figure 6:**
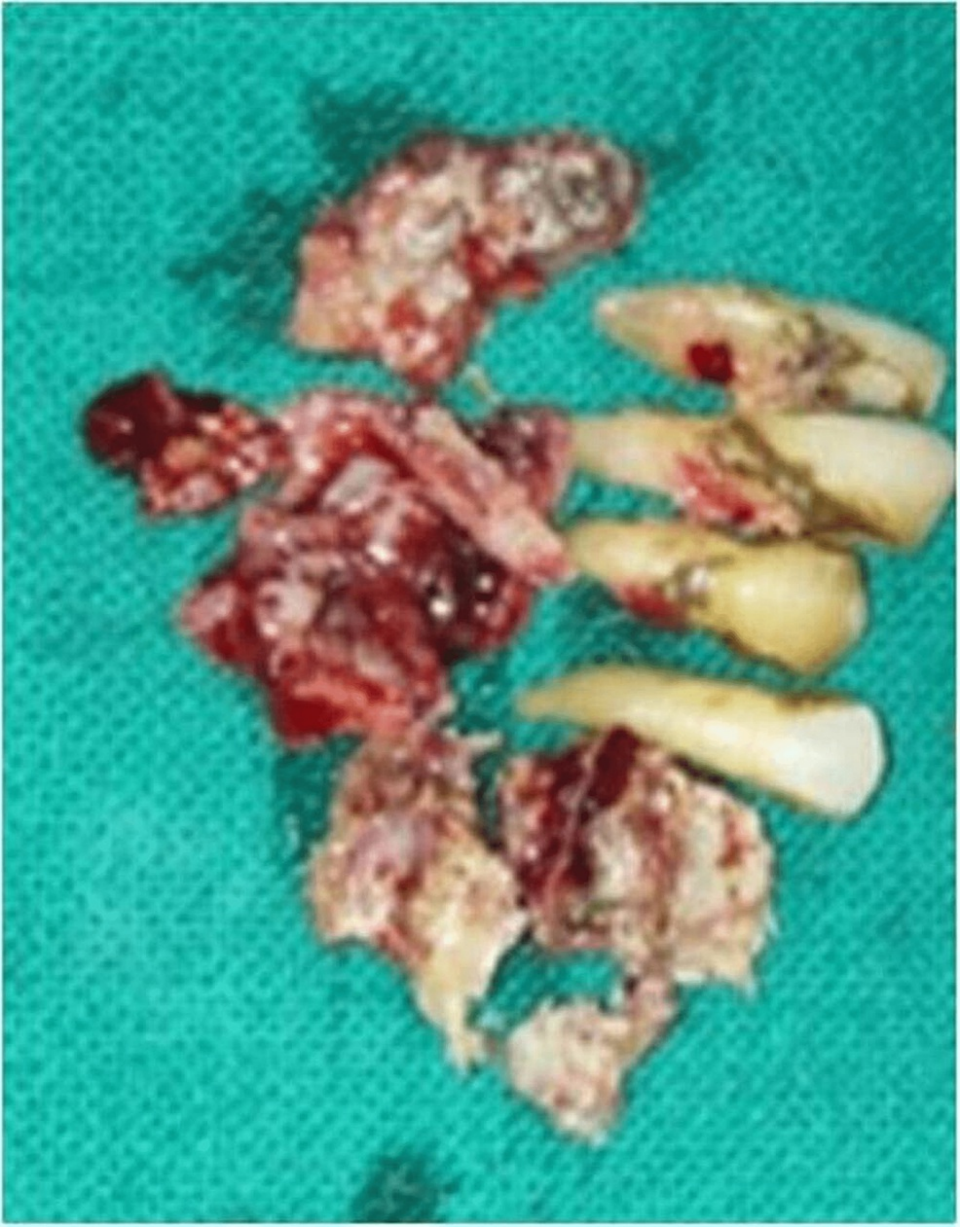
Specimen grossing picture after open surgical debridement. Teeth were removed along with necrotic tissue.

**Figure 7:**
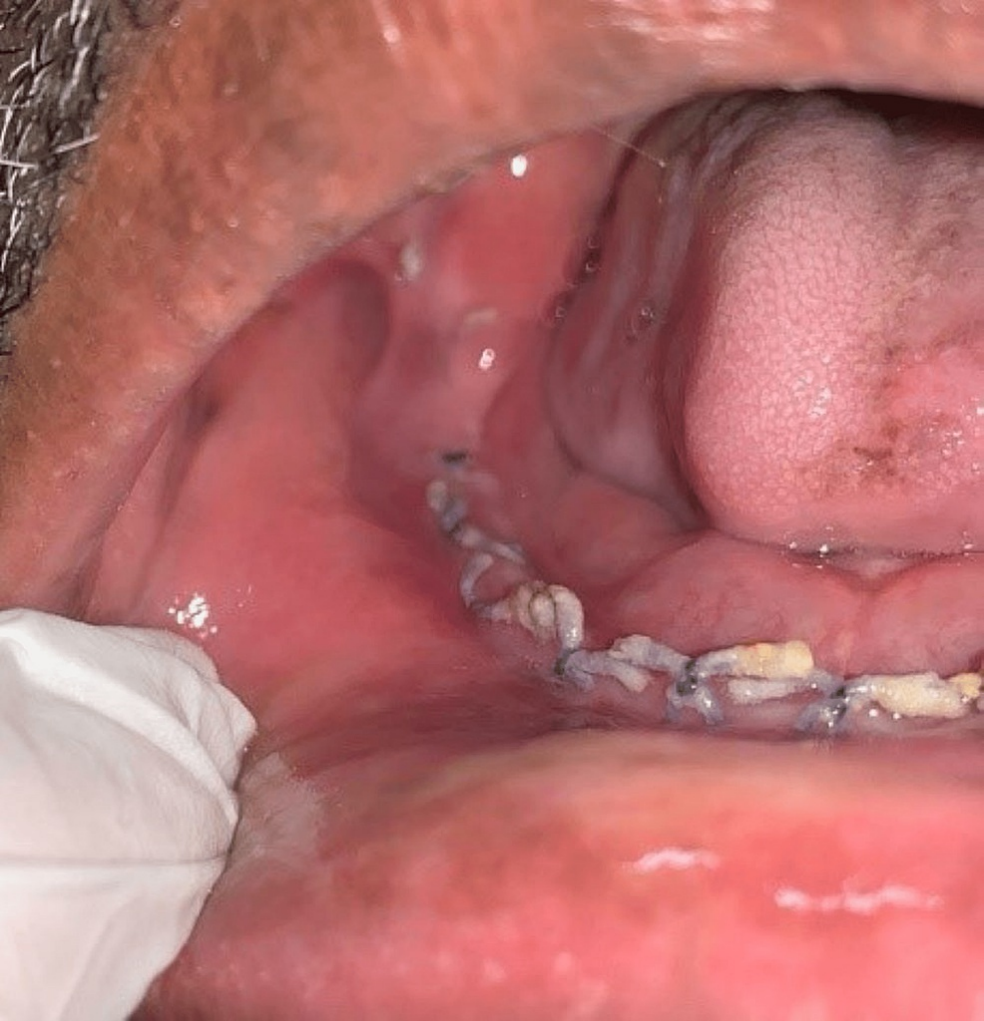
Post-operative view shows uneventful healing of the surgical site.

According to the institution's procedure, the patient was promptly put on liposomal amphotericin B (5 mg/kg body weight for 21 days) and posaconazole (300 mg delayed-release tablets twice a day for one day followed by 300 mg daily), and an open surgical debridement of the mandible was scheduled. An intraoral vestibular approach was used to perform a marginal mandibulectomy under rigorous aseptic procedure on the right side of the afflicted mandible (Figures 5-7). After discharge, posaconazole 300 mg once daily was continued for two months. Patient’s follow-up was done during the uncomplicated post-operative period. The patient was healthy and showed no evidence of a recurrence three months after the follow-up visit.

## Discussion

**Figure 8:**
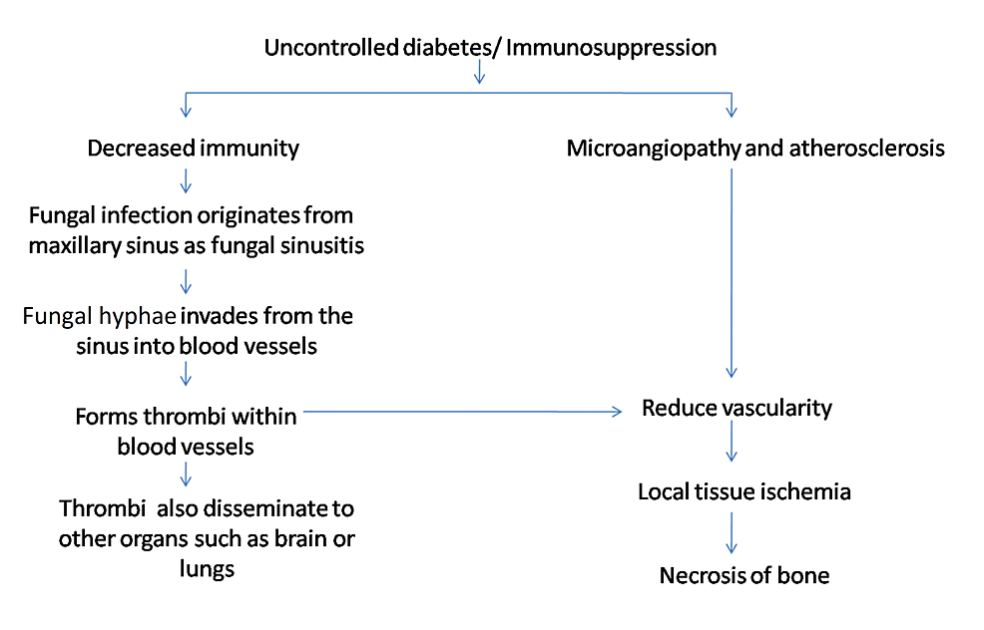
Pathogenesis of mucormycosis.

Mucormycosis, one of the most deadly and fulminating fungal infections in humans, starts typically in the nose and paranasal sinuses. The fungus enters the blood vessels through angioinvasion of the descending palatine artery and internal maxillary artery. In blood arteries, mucor hyphae create thrombi that reduce tissue vascularity and result in necrosis. Furthermore, because of the elevated level of angiotensin II, thrombosis is more likely in COVID-19 patients. Due to its direct atherosclerotic effects on the vascular wall, angiotensin II is shown to be prothrombotic. These effects include increased inflammation, endothelial dysfunction, oxidative stress, endothelial cell and vascular smooth muscle cell migration, growth, and proliferation [5]. Figure 8 summarizes the etiology of mucormycosis.

The cytokine storm, an auto-amplifying syndrome of proinflammatory cytokines, is also brought on by SARS-CoV-2. Endothelial cell vascular damage is brought on by an increase in a number of proinflammatory cytokines, such as tumor necrosis factor-alpha (TNF-alpha) and interleukin (IL)-2R, IL-6, IL-8, and IL-10. Numerous of these cytokines exhibit prothrombotic properties. The lungs, spleen, brain, stomach, and peripheral vasculature experience microvascular and macrovascular thromboembolic events as a result [6].

Patients with rhinocerebral mucormycosis may complain of face discomfort and/or coloring, swelling, nasal or oral pus discharge, the appearance of a reddish-black nasal septum and turbinate, paresthesia, proptosis, toothache, and mobility of teeth [3-8].

As the illness worsened, sinus tracts and tissue sloughing might emerge, and necrosis could spread to the paranasal sinuses and orbital cavities. As a result of the infection in the nasal cavity or paranasal sinuses via palatal vessels, the oral cavity involvement usually begins as palatal ulceration or necrosis and subsequently as perforation of the palate. Clinically, phycomycosis affecting the maxillary sinus might manifest as a tumor in the maxilla that resembles antrum cancer. Once the orbit is implicated, it could exhibit orbital cellulitis [3-8].

The differential diagnoses include bacterial necrotizing infections, aspergillosis and other fungi diseases, cancer, rhinoscleroma, TB, syphilis, and other granulomatous disorders. Finding the invasive saprophytic fungus in the afflicted tissue is necessary for a definitive diagnosis as it dwells in the nose and mouth [2,9].

Amphotericin B, posaconazole, or isavuconazole are used as antifungal medications in primary care, combined with surgical debridement of necrotic tissue. The patient's underlying predisposing disease process, the amphotericin B injection, and early aggressive surgical debridement proved to be the three most important determinants affecting survival. Survival improved to 78% in patients who underwent broad surgical debridement as opposed to 57.5% in those who did not. Arterial thrombosis prevents amphotericin B from getting to the fungus because it thrives in necrotic tissue. Consequently, surgical debridement eliminates an infection source that is resistant to systemic therapy. Polyene antifungal drug's (L-AMB) liposomal formulation has less nephrotoxicity and is prescribed during the initial course of mucormycosis treatment. L-AMB was recommended for our patient as well [2,10]. In diabetic patients, survival can be increased by actively treating the underlying medical conditions, volume replacement, and acidity correction. Treating the immunosuppression due to steroid therapy, hematologic cancer, burns, chronic renal failure, or other immunosuppression syndromes is challenging, and it is associated with a poor survival rate [10].

## Conclusions

Although rare and often misdiagnosed, mandibular mucormycosis might be reported to maxillofacial surgeons. This case report aimed to shed light on mandibular mucormycosis, its hypothesized etiopathogenesis, and the recommended course of therapy. This lethal disease can be prevented or reduced in frequency with early intervention and competent medical and surgical treatment with full debridement.

## References

[1] Singh AK, Singh R, Joshi SR, Misra A (2021). Mucormycosis in COVID-19: a systematic review of cases reported worldwide and in India. Diabetes Metab Syndr.

[2] Kwak EJ, Kim DJ, Nam W, Park W (2020). Mucormycosis in the Jaw: a report of 2 cases and literature review. Oral Health Prev Dent.

[3] Shivhare P, Parihar A (2021). Fungal infection of oral cavity. Textbook of Oral Medicine and Radiology. Second Edition.

[4] Oswal NP, Gadre PK, Sathe P, Gadre KS (2012). Mucormycosis of mandible with unfavorable outcome. Case Rep Dent.

[5] Castro RA, Frishman WH (2021). Thrombotic complications of COVID-19 infection: a review. Cardiol Rev.

[6] Hanff TC, Mohareb AM, Giri J, Cohen JB, Chirinos JA (2020). Thrombosis in COVID-19. Am J Hematol.

[7] Bhanumurthy L, Krishna PS, Sekhar P, Makesh Raj LS (2021). Post coronavirus disease mucormycosis involving the mandible: a case report with brief note on literature. J Oral Maxillofac Pathol.

[8] Verma M, Sharma R, Verma N, Verma K (2020). Rhinomaxillary mucormycosis presenting as palatal ulcer: a case report with comprehensive pathophysiology. J Oral Maxillofac Pathol.

[9] Aggarwal P, Saxena S, Bansal V (2007). Mucormycosis of maxillary sinus. J Oral Maxillofac Pathol.

[10] Aras MH, Kara MI, Erkiliç S, Ay S (2012). Mandibular mucormycosis in immunocompromised patients: report of 2 cases and review of the literature. J Oral Maxillofac Surg.

